# Supplementary figures and images for: Species specific marker genes for systemic defence and stress responses to leaf wounding and flagellin stimuli in hybrid aspen and silver birch
Source: PLoS One. 2026 Mar 12;21(3):e0344803. doi: 10.1371/journal.pone.0344803 (PMC12981503; doi:10.1371/journal.pone.0344803)

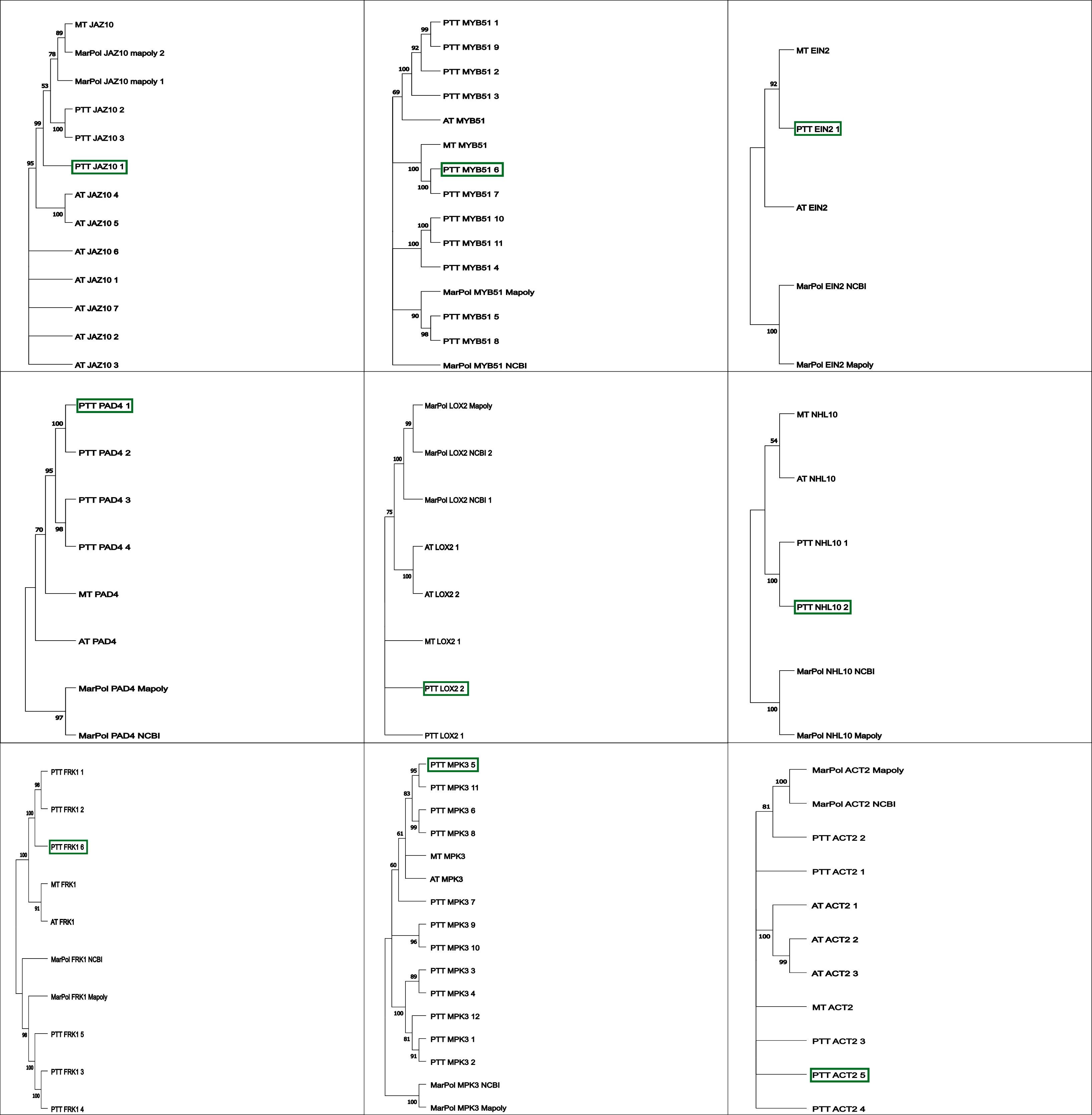

Supplement: S1 Fig — Phylogeny was based on translated protein sequences of JAZ10, MYB51, EIN2, PAD4, LOX2, NHL10, FRK1, MPK3, and reference gene ACT2 from Populus tremula x tremuloides (PTT), Arabidopsis thaliana (AT), Medicago truncatula (MT), Marchantia polymorpha (MarPol). In case of multiple spliced variants, all sequences were included for tree construction using the Maximum likelihood method. Green box denotes the homolog used for primer design in the study. (TIFF) [file pone.0344803.s004.tiff]

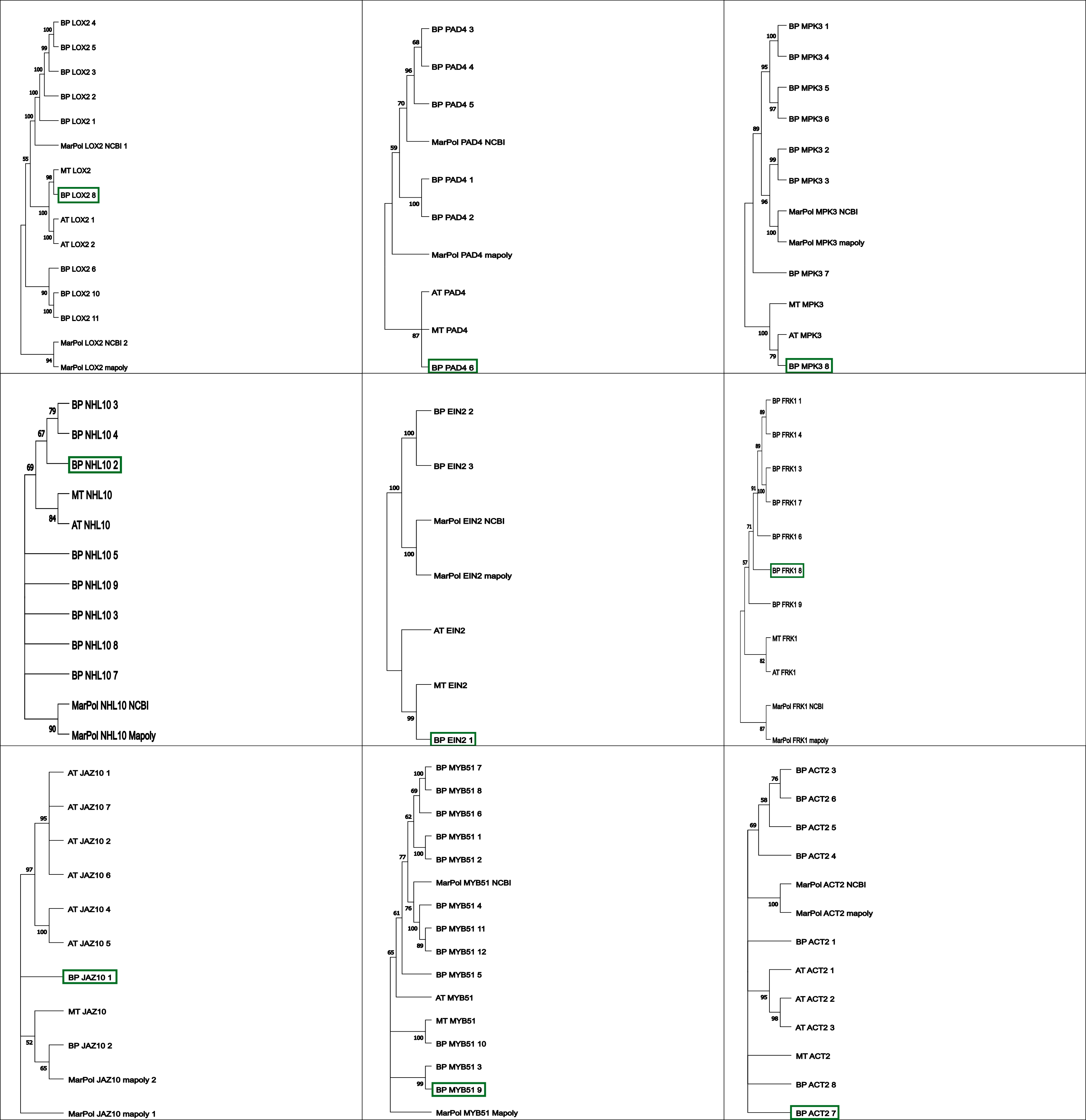

Supplement: S2 Fig — Phylogeny was based on translated protein sequences of JAZ10, MYB51, EIN2, PAD4, LOX2, NHL10, FRK1, MPK3, and reference gene ACT2 from Betula pendula (BP), Arabidopsis thaliana (AT), Medicago truncatula (MT), Marchantia polymorpha (MarPol). In case of multiple spliced variants, all sequences were included for tree construction using the Maximum likelihood method. Green box denotes the homolog used for primer design in the study. In cases where multiple potential homologues clustered near MT and/or AT sequences, the candidate with the highest homology scores (provided by the BLAST tools used in each case) as well as with similar results in the reverse BLAST procedure were chosen. (TIFF) [file pone.0344803.s005.tiff]

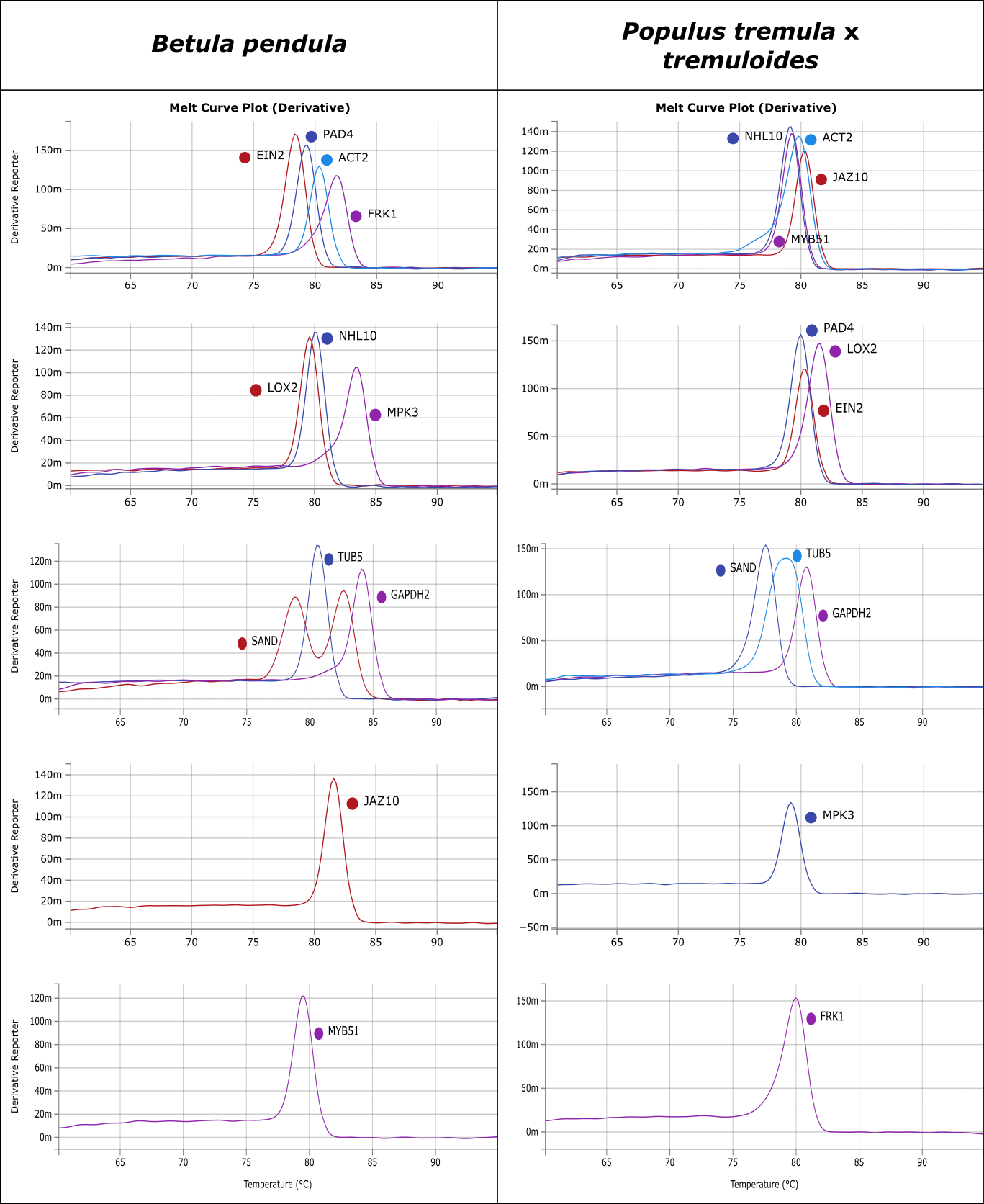

Supplement: S3 Fig — (TIFF) [file pone.0344803.s006.tiff]

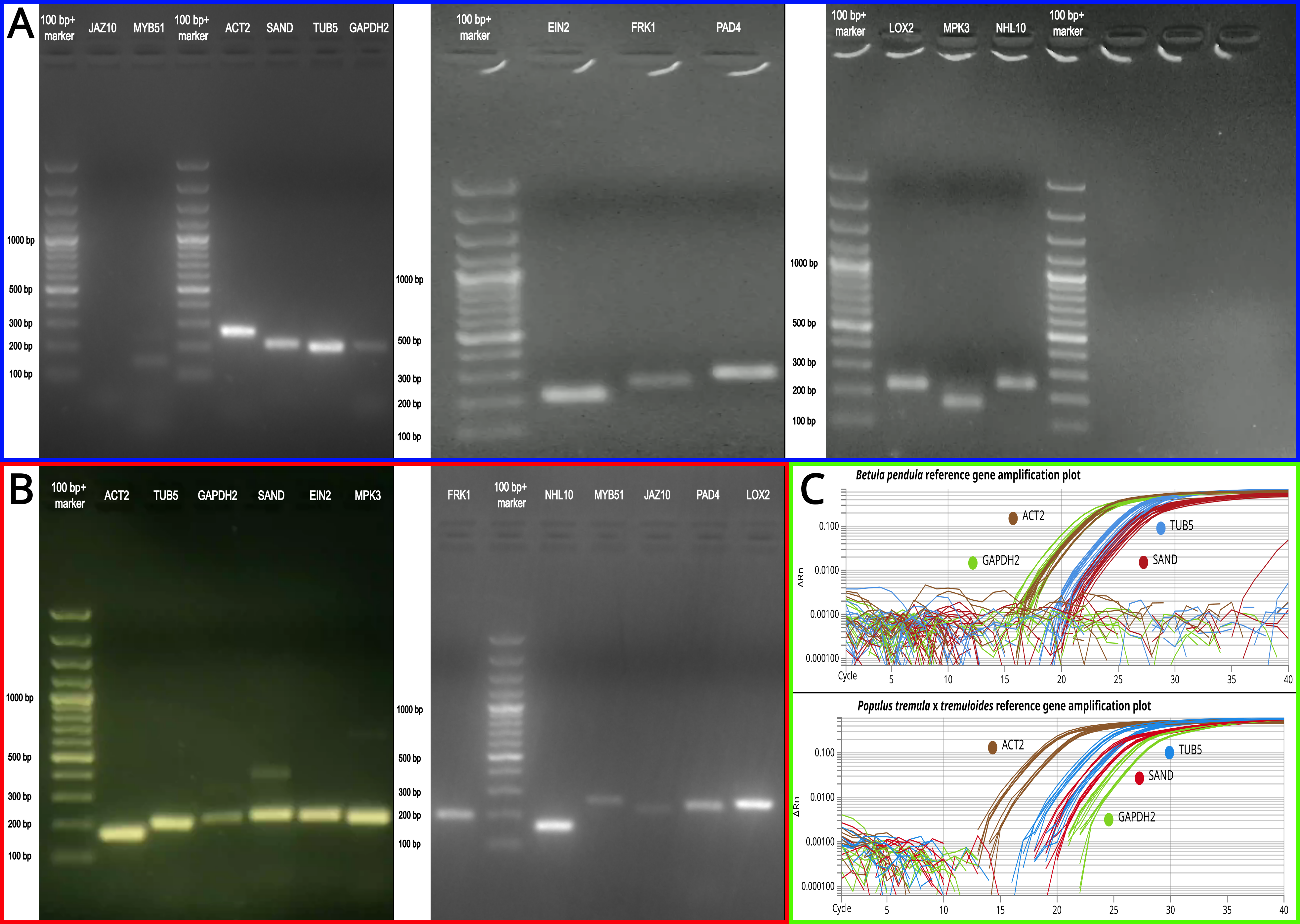

Supplement: S4 Fig — 35-cycle qPCR amplicons were used to check for primer specificity, target size and absence of double bands (A-B). deltaRn indicates the intensity of SYBR signal corresponding to target amplification during 35 qPCR cycles. Reference gene ACT2 was selected based on earlier amplification and uniform expression across the different stress treatments in the qPCR amplification curve (C). (TIFF) [file pone.0344803.s007.tiff]

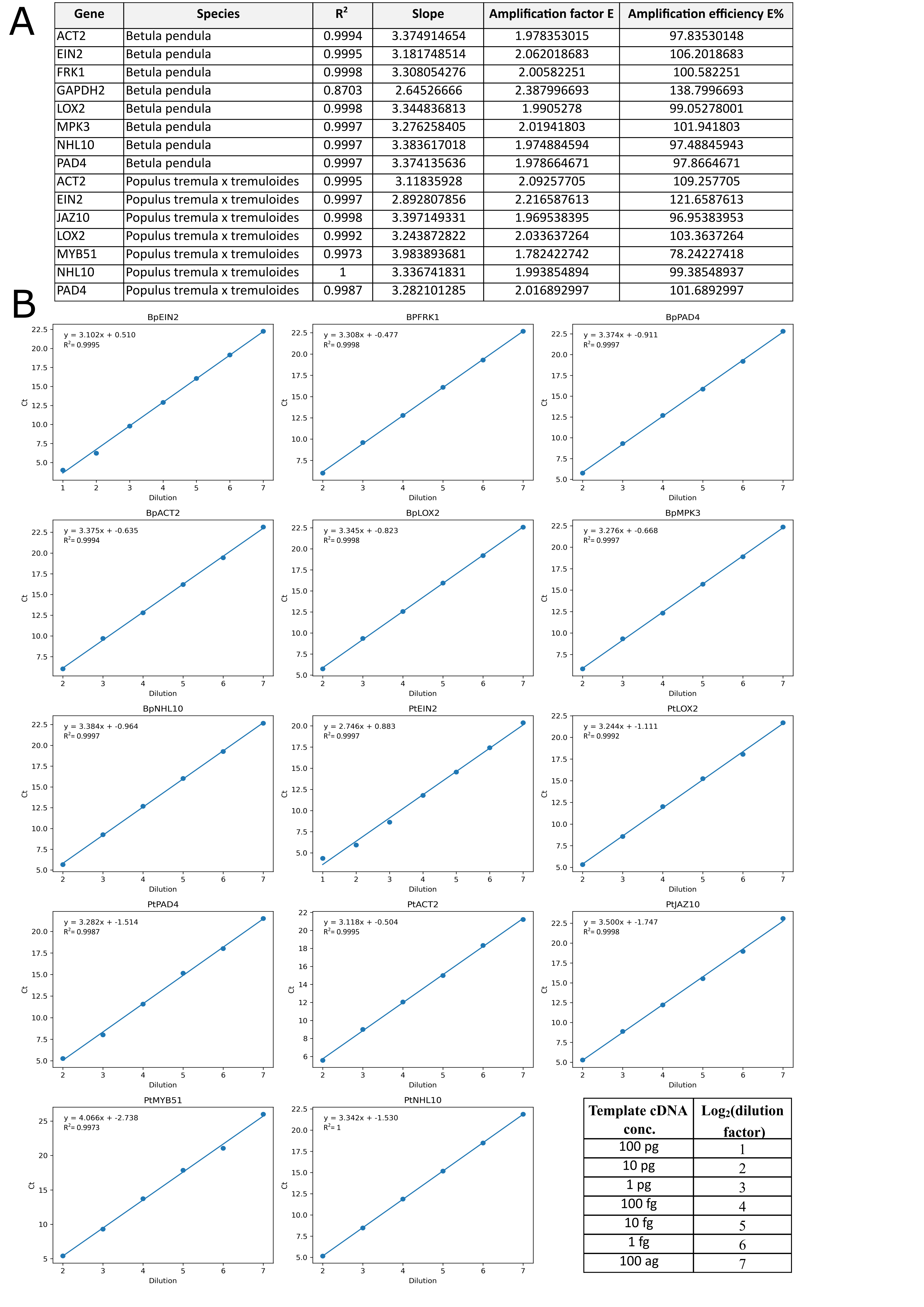

Supplement: S5 Fig — (A) All stress marker genes used in this study are listed with their amplification efficiency (E%) and amplification factor (E) used for calculating ΔCt values. Calculation of E% is based on formula E(%)=(−110Slope−1)×100. (B) Slope values are based on the regression lines for the Ct values for amplification of 10-fold dilution series of the template DNA, starting from 100 pg. (PNG) [file pone.0344803.s008.png]

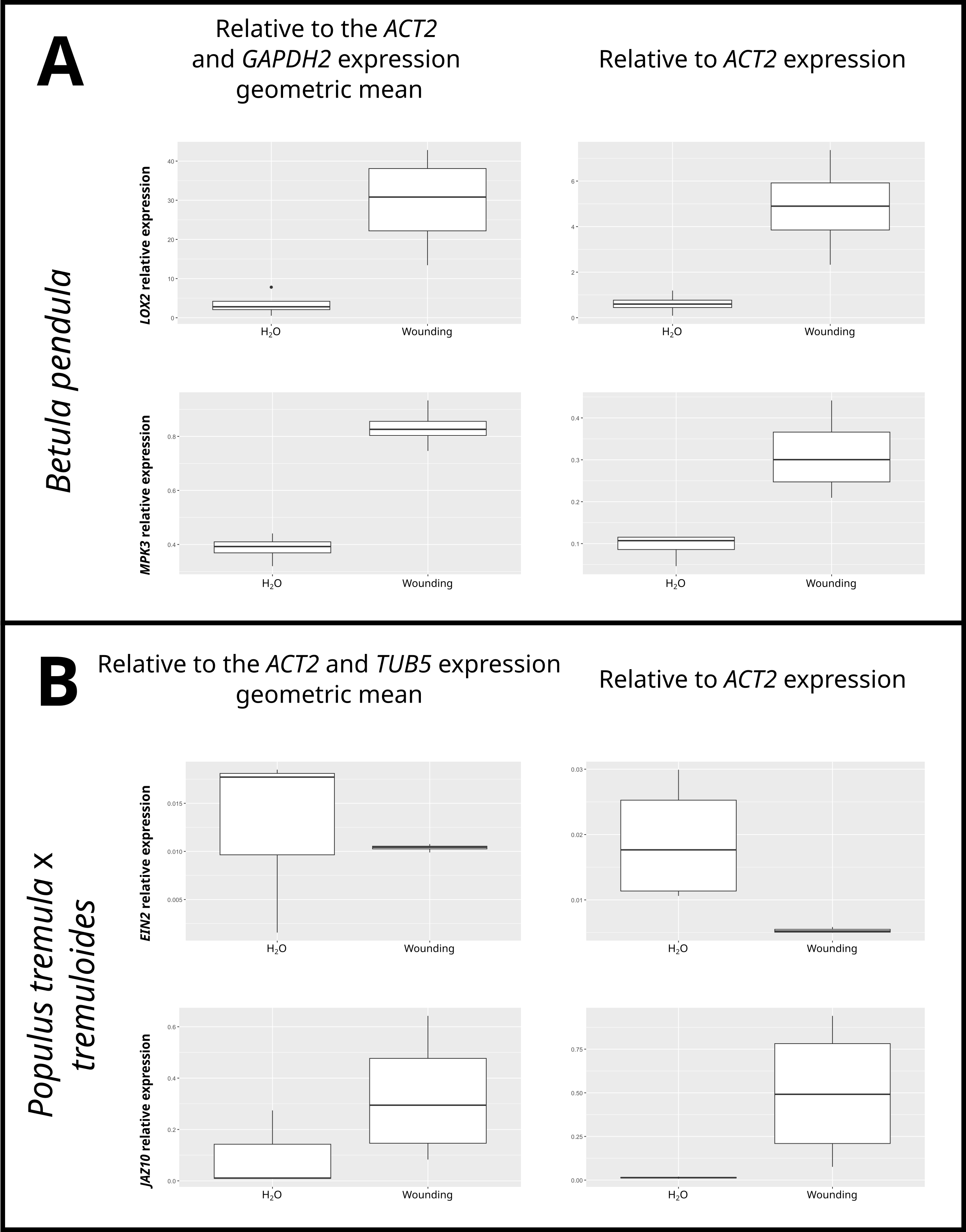

Supplement: S6 Fig — To test whether ACT2 is the optimal reference gene for defence gene expression testing, qPCR was carried out with an additional potential reference gene - GAPDH2 and TUB5 for B. pendula and P. tremula x tremuloides respectively and the relative expression of select genes and treatments was calculated relative to the geometric mean of both potential reference genes and compared to original expression data (only ACT2 as a reference). Geometric mean of two selected reference genes is calculated with the formula Eref1Ct(ref1) × Eref2Ct(ref2)EtestCt(test), where Eref – primer efficiency of reference genes 1 and 2, Etest – primer efficiency of test gene, Ct(ref) – Ct value of reference genes 1 and 2, Ct(test) – Ct value of the test gene. While both tested hybrid aspen genes showed differences in statistical significance, the expression tendencies remained similar in both cases. Difference in result significance could potentially be explained by the fact that the original models, which included all treatments, used ANOVA and/or Kruskal-Wallis tests, while the present experiment used t and Wilcoxon signed ranked tests, since only two groups were compared. (TIFF) [file pone.0344803.s009.tiff]

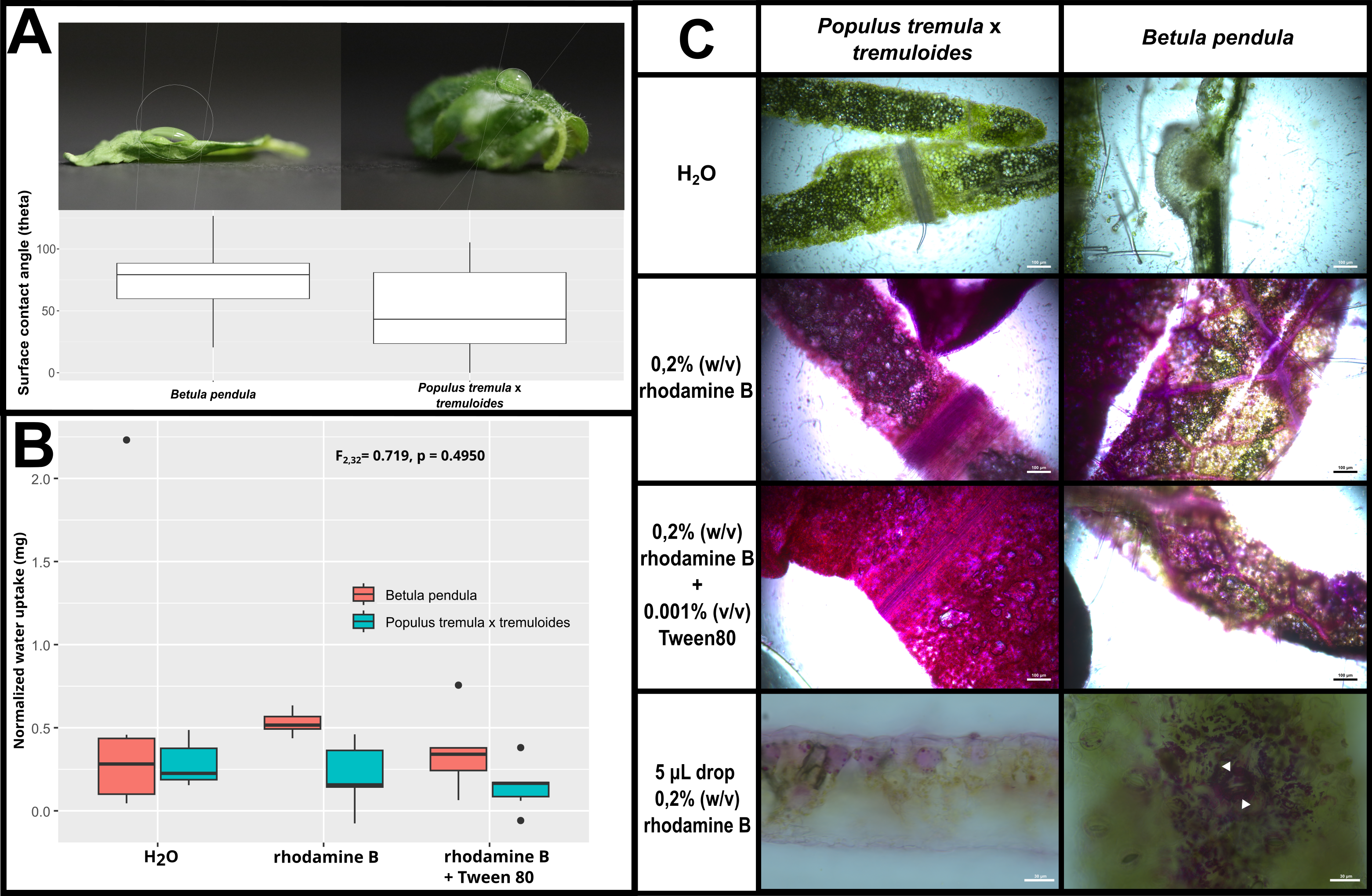

Supplement: S8 Fig — (A) Microscopy of B. pendula and P. tremula x tremuloides leaves treated with rhodamine B. Three different treatments were used – submergence in rhodamine B solution (Rb), submergence in rhodamine B solution with added Tween 80 (Rb + Tween 80), a single drop (5 μL) of rhodamine B solution on the abaxial leaf surface (Rb drop) and a control group of a leaf submerged in water (H2O). Submergence photos were taken with 10x magnification; Rb drop photos with 40x. All photos were taken 3 hours post treatment. White triangle on the P. tremula x tremuloides Rb drop photo indicates a stoma – a potential entry point for Rb solution (and water) infiltration. B – Difference in water uptake 3 hours post treatment between different treatments and both tree species. No significant difference was observed between different treatments, while B. pendula water uptake was significantly higher than P. tremula x tremuloides. Regardless, all experimental groups demonstrated water uptake through the leaf surface. C – results of the leaf surface wettability experiment. The wettability of the leaf surface for both tree species was carried out, by determining the surface contact angle of a water drop on the surface of a leaf (shown in the photographs). The median value of the surface contact angle of the water droplet was lower than 90 degrees, indicating the wettability of the leaf surface for both species. (TIFF) [file pone.0344803.s011.tiff]
