# Supplementary material for: Species specific marker genes for systemic defence and stress responses to leaf wounding and flagellin stimuli in hybrid aspen and silver birch
Source: PLoS One. 2026 Mar 12;21(3):e0344803. doi: 10.1371/journal.pone.0344803 (PMC12981503; doi:10.1371/journal.pone.0344803)
Supplement: S1 File — (DOCX) [file pone.0344803.s012.docx]

**Detailed description of the modified CTAB extraction protocol used for total RNA extraction**

Prior to the extraction, all labware was treated with RNaseZap™ tissues to eliminate any traces of contaminants. The 1.5- and 2-mL test tubes were submerged in 0.1% DEPC solution for 24 h and autoclaved three times at 121 °C for 40 min, using saturated steam with pressure of at least 103 kPa (15 psi), to ensure the degradation of DEPC. Leaf samples were homogenized in liquid nitrogen using a pestle and mortar before transferring the material into 2 mL test tubes with 900 μl CTAB extraction buffer (2-cetyltrimethylammonium bromide; 0.1M Tris-HCl (pH 8); 1.4M NaCl; 20mM Ethylenediaminetetraacetic acid (EDTA) (pH 8); 2% polyvinylpolypyrrolidone (PVPP)) and 100 μl of β-mercaptoethanol). The mixture was shaken for 30 s and incubated in a thermoblock for 10 min at 65 ℃. After incubation, 800 μl of chloroform were added to the mixture, which was followed by repeated shaking for 30 s. The mixture was then centrifuged for 10 min at 4 ℃ and 10’000 rpm.

The supernatant was transferred to a new 2 mL test tube together with 800 μl of a phenol/chloroform/isoamyl alcohol mixture (50:49:1) and shaken for 30 s. Samples were then centrifuged for 10 min at 10’000 rpm and 4 ℃ and the supernatant was transferred to a new 2 mL test tube together with an equal volume of a chloroform and isoamyl alcohol mix (24:1). The samples were then shaken for 30 s and centrifuged at 10’000 rpm at 4 ℃. The supernatant was transferred to a new 1.5 mL test tube together with a third of the supernatant volume of 8M LiCl. Subsequently the samples were incubated for 24 h at -20℃, after which they were centrifuged for 20 min at 4 ℃ and 10000 rpm.

The sediment pellets were then washed with 1 mL 96% and 70% ethanol and centrifuged for 5 min at 10000 rpm. After centrifugation, ethanol was discarded, and the samples were dried at room temperature. The sediment pellets were then dissolved in 15 μl of 1x TE buffer with 1 μl of 1u/μl RNasin® Plus ribonuclease inhibitor solution (Promega). The mix was then treated with RQ1 Rnase free Dnase kit (Promega) according to manufacturer instructions. This process was followed by RNA extraction using a phenol/chloroform/isoamyl alcohol mixture (50:49:1). For convenience, 230 μl of 1x TE buffer were added to 10 μl of the sample. The phenol/chloroform/isoamyl alcohol mixture was added at a ratio of 1:1 to the sample solution.

After the extraction, RNA was sedimented by adding 0.1 volume of 3M sodium acetate and 2 volumes of 96% ethanol, which was followed by 24 hours of incubation at -20 ℃. Following the incubation period, the mixture was centrifuged for 15 min at 13’000 rpm and 4 ℃ and subsequently washed with 70% ethanol. The RNA sediment pellets were dried at room temperature and dissolved in 20 μl of TE buffer together with 1 μl of RNase inhibitor. The mRNA samples were stored at -80 ℃. Total mRNA sample concentration and purity were measured using a “NanoDrop 2000” spectrophotometer (Thermo Scientific).
