# Supplementary material for: Species specific marker genes for systemic defence and stress responses to leaf wounding and flagellin stimuli in hybrid aspen and silver birch
Source: PLoS One. 2026 Mar 12;21(3):e0344803. doi: 10.1371/journal.pone.0344803 (PMC12981503; doi:10.1371/journal.pone.0344803)
Supplement: S2 File — (DOCX) [file pone.0344803.s013.docx]

**A representative selection of R studio code lines used in data analysis**

# Libraries----

# Data import

library(readxl) # For importing excel files

# Statistics

library(car) # For Levene test

library(dunn.test) # For Dunn post-hoc test

# Visualization

library(ggpubr) # For qqplots

library(qqplotr) # For qqplots

library(pheatmap) # For heatmaps

library(ggplot2) # For plot adjustments

# Linear model and data manipulation----

library(plyr) # For data manipulation

library(emmeans) # For estimated marginal means

library(multcomp) # For comparisons

library(multcompView) # For compact letter displays

library(tidyverse) # For workflow

# Data import----

data_bp <- read_excel("qpcr_data_both_trees.xlsx", sheet=1)

is.factor(data_bp$Treatment)

data_bp$Treatment <- as.factor(data_bp$Treatment)

data_bp_ein2_4h <- data_bp %>% # For group comparisons

  filter(Gene=="EIN2") %>%

  filter(Timepoint=="4h")

Heatmap_BP_dati_4h<- read_excel("qpcr_data_both_trees.xlsx", sheet=2) %>% # For heatmaps

  filter(Timepoint=="4h") %>%

  group_by(Gene, Treatment) %>%

  select(Treatment, Gene, Value) %>%

  pivot_wider(names_from = Gene, values_from = Value)

# Data vizualization----

bp_ein2_4h_boxplot <- ggplot(data_bp_ein2_4h,aes(Treatment, Value)) + geom_boxplot()

bp_ein2_4h_boxplot

# Homogeneity of variance----

leveneTest(Value ~ Treatment, data = data_bp_ein2_4h)

# Group comparison - ANOVA----

ein2_4h_aov <- aov(Value ~ Treatment, data = data_bp_ein2_4h)

summary(ein2_4h_aov)

# Checking the distribution of residuals----

ggplot(data.frame(resid = residuals(ein2_4h_aov)), aes(sample = resid)) +

  geom_qq_band() + stat_qq_line() + stat_qq_point() +

  labs(x = "Theoretical quantiles", y = "Sample quantiles")

shapiro.test(residuals(ein2_4h_aov))

# Post hoc test----

ein2_tukey <- TukeyHSD(ein2_4h_aov)

ein2_tukey

# If residuals were non-normal - Kruskal-Wallis test----

ein2_4h_krusk <- kruskal.test(Value~Treatment, data = data_bp_ein2_4h)

ein2_4h_krusk

# Alternative post-hoc test - Dunn test---

ein2_dunn <- dunn.test(data_bp_ein2_4h$Value, data_bp_ein2_4h$Treatment, method = "bonferroni")

# Heatmap----

BP4=as.matrix(Heatmap_BP_dati_4h[,-1])

row.names(BP4)=Heatmap_BP_dati_4h$Treatment

BP4_heatmap <- pheatmap(BP4, fontsize=14, main = "BP 4 hours")

BP4_heatmap

# Linear model----

birch<-as.data.frame(read_xlsx("qpcr_data_both_trees.xlsx", sheet=1)) #additional column specifying species should be added

aspen<-as.data.frame(read_xlsx("qpcr_data_both_trees.xlsx", sheet=3)) #additional column specifying species should be added

data<-rbind(birch, aspen)

rm(birch, aspen)

data$id<-paste(data$Species,data$Gene, data$Timepoint, sep="_")

contr<-ddply(data, .(id), summarize,

             control_mean=mean(Value[Treatment=="Control (H2O drop)"]))

data<-join(data,contr, by="id", type="left" )

rm(contr)

data$logfold1<-log(data$Value/data$control_mean,2)

logfold<- data[data$Treatment1!="Control (water)",]

mod<-lm(logfold~Treatment*Gene*Timepoint,

        offset = control_mean,

        data = logfold[logfold$Species=="Silver birch",])
