# Supplementary material for: Species specific marker genes for systemic defence and stress responses to leaf wounding and flagellin stimuli in hybrid aspen and silver birch
Source: PLoS One. 2026 Mar 12;21(3):e0344803. doi: 10.1371/journal.pone.0344803 (PMC12981503; doi:10.1371/journal.pone.0344803)
Supplement: Attachment — Submitted filename: Respone to reviewers PlosOne_Feb 2026.docx [file pone.0344803.s016.docx]

**PONE-D-25-43089**

Species specific marker genes for systemic defence and stress responses to leaf wounding and flagellin stimuli in hybrid aspen and silver birch

Dear editor,

We are grateful for careful consideration and evaluation of our revised manuscript **PONE-D-25-43089** for publication in *PlosOne*. Following the feedback and comments from the reviewers and editor, we here resubmit our revised manuscript with the following corrections to our manuscript:

*1. Combined treatment limitation*

*Please add one clear sentence in Results or Discussion that the absence of a wounded plus water control means the combined treatment cannot distinguish synergy or additivity from enhanced entry at the wound. Keep any language about interaction effects cautious.*

In line 330-332 we added a new sentence to explicitly state ‘’However, without a wound plus water control, the combined treatment does not allow clear separation of synergistic or additive interactions from potential effects of enhanced entry at the wound.’’ This is corroborated by further statements in discussion (lines 471-477) from the first round of revisions, acknowledging the benefits from including the water + wound control in future experiments.

*2. Circadian timing note*

*Please add one sentence in Discussion to state whether time of day was controlled or recorded. If not controlled, note that diurnal variation may contribute to some differences.*

We thank reviewer for suggesting to include the missing detail. We performed leaf stimulation at the same time of the day for all treatments. Therefore, we included a new sentence in the methods section: (line 140-141) ‘’To minimize potential diurnal variation in gene expression, all stimuli were applied at noon (12:00), 4h after the onset of grow chamber lighting. ‘’

*3. Standard curve reporting*

*You report slopes for the qPCR dilution curves. Please add the corresponding R squared values for each assay in the supplement or state the range across assays. This helps readers judge amplification efficiency quality.*

We have added the R-squared values and the regression equations for each dilution in Supplemental Figure 5.

*Minor comments*

*– If space allows, indicate n on main figure panels that show distributions. If not, the legend statement on replication is acceptable.*

We have included the number of replicates in Figure 1. We added an extra sentence ‘’Each boxplot represents the average of 4 biological replicates. The total number of replicates for statistical analysis across treatments is indicated in each panel. ‘’ in the legend. We added a sentence in the legend of Figure 2: “Each rectangle represents the average of 4 biological replicates (3 in the cases of silver birch 4h flg22 LOX2, MPK3, NHL10; hybrid aspen 24h flg22 EIN2, LOX2, PAD4 and 24h flg22+w MYB51).”

For the ease of tracking changes in the manuscript, we include the file *xxx_track changes.docx.* **The final version** with all track changes accepted is also enclosed and uploaded to the online submission system.

We hope that the improvements presented herein will be sufficient to address the points raised by the reviewer and provide readers with new in-depth information for better interpreting the results presented herein. We are looking forward to your final decision.

Sincerely,

Dr. Zigmunds Orlovskis

**Reviewer #1:** 
1. Abstract nicely written, but could include one key result (e.g., “LOX2, MPK3, and EIN2 were early wounding markers in birch; JAZ10 and EIN2 responded in aspen to combined treatments”) for a stronger takeaway.

We thank the reviewer for the suggestion. We added a sentence in the abstract to highlight the key result of our study (lines 34-36): ‘’We identified MPK3, LOX2 and EIN2 as potential early response wounding markers in silver birch and JAZ10 with EIN2 as potential response markers for the combined effect of flagellin and wounding in hybrid aspen’’. Moreover, in combination to similar changes suggested by reviewer 2, we have harmonized the description of the study aim and take-home message across the abstract, introduction and the Conclusion.

2. Consider separating ecological background from molecular aims for clarity.

We have streamlined the introduction to add a bit more separation between the ecological background and economic value of hybrid aspen and silver birch in Northern economies in the background of the anticipated climate change (lines 46-73). We have identified the knowledge gap in understanding the molecular stress responses in high yielding tree clones (lines 73-88) and defined the molecular aim of the current study (lines 89-93) accordingly. We believe that the aims and rationale should now be clear and understandable to the reader and are separated in distinct paragraphs.

3. Methodology is very detailed and good for reproducibility. Could move the long RNA extraction protocol to Supplementary Methods and summarize the key steps in the main text. While the methodological development is valuable, the study mainly reports expression differences without deeper mechanistic exploration (e.g., hormone quantification, protein activity, imaging of ROS).

We thank the reviewer for the proposition. We moved the full RNA extraction protocol to the supplement section and added a shortened summary of the key steps in the main text (lines 144-149) as follows: “Total RNA was extracted following a modified CTAB extraction protocol from Rubio-Piña and Zapata-Pérez (2011) adapted for use with woody plant tissue (detailed protocol in supplementary methods). Reverse transcription reaction mix was prepared using the Thermo Scientific Maxima First Strand cDNA synthesis kit according to manufacturer instructions using 500 ng of total RNA. The reaction was carried out using a BIO-RAD T100TM thermal cycler and the reaction mix was subsequently diluted to 800 μl.” The detailed and extended RNA extraction protocol is now available as the **Supplementary File 1**.

4. Include one representative tree figure in the main text to visually confirm gene homology.

We thank the reviewer for the suggestion. We have generated a new figure (**Figure 1**) featuring the phylogenetic trees of the identified *EIN2* homologues in both tree species (**Figure 1A**). We included a description for the figure in the main text, lines 266-270. As suggested in the comment Nr. 5 from reviewer #2, we also decided to promote the boxplots for *EIN2* expression to this figure (**Figure 1B**) to represent an example result for finding a homologue and determining the expression of EIN2 under different time points and stress conditions. This logically tells a story about the methodological approach in identifying tree stress gene homologues using EIN2 as an example for all other marker genes tested in our study. This is then followed by fold change analysis of all stress responsive markers in the main **Figure 2** (previously **Figure 1**) as well as potential primer divergence of selected stress markers as **Figure 3**. These changes satisfy comments #4 & #6 by reviewer #1 as well as comment comment # 5 from reviewer #2, provide a distinct Figure for each sub-section of the Results section. We believe that this adds overall value and depth to our study.

5. Figure 1: It’s unclear how fold changes were normalized (ΔΔCt formula not shown in text). Add a legend clarifying “relative to ACT2 and to control.”

We acknowledge that we can improve the clarity for fold change calculations description in our manuscript. To this end, we have added the missing details in the methods section (line 165-174) by explaining that the fold change (ΔΔCt) was obtained by dividing the ΔCt for each treatment with the average ΔCt of the water control. The ΔCt for each test gene was obtained by normalizing gene expression to ACT as reference gene. Moreover, ΔCt takes into consideration primer efficiency. For clarity, we included the exact formula and explanation both in the methods section and in the Figure 2 legend as well.

6. Expand discussion on why LOX2 was inactive in aspen but active in birch, possibly due to promoter divergence or JA pathway suppression.

We appreciate the thoughtful comment by the reviewer. *LOX2* and *EIN2* were among two genes that showed both species- and time-specific expression changes in response to wounding and flg22 treatments. Therefore, to assess whether gene regulatory sequences could, in part, we implicated in the observed expression patterns, we performed an additional *in silico* analysis of LOX2 promoter sequences from both tree species to assess the sequence conservation and characterise TCP transcription factor (TF) binding sites within 5k region upstream UTR and transcription start site. We added the new set of results as **Figure 3** of the main text and included a new results **section 3.3** to describe the results supporting the hypothesis that promoter divergence and putative differences in transcription factor binding site presence could be implicated in species specific responses to mechanical wounding and immunogenic elicitors.

Intriguingly, the LOX2 promoter in hybrid aspen displayed sequence divergence from silver birch compared to much greater conservation and sinteny of conserved promoter islands with an evolutionary more closely related tree species such as *Populus trichocarpa*. Since TCP transcription factors include well characterised JA synthesis regulators in plant immunity and wound responses (https://[doi.org/](https://doi.org/10.1105/tpc.104.026609)10.1016/j.tplants.2015.01.005), we additionally decided to retrieve the known binding motifs of *LOX2* positive regulator TCP4 and its negative regulators TCP20 and TCX8 (Noh et al., 2021). When we searched for their binding motifs within the *LOX2* promoter region of both tree species, we identified tree species specific presence and distribution of the binding sites. Furthermore, although TCP4 is known positive *LOX2* regulator for wound-induced JA synthesis, we also found its putative binding sites within the promoter of *EIN2* in silver birch but not in aspen or Arabidopsis, corroborating the possibility of species specific gene responses in evolutionary diverse species.

While we could not provide experimental data on transcription factor binding to wound & flg22 responsive genes, the *in silico* analysis provided a valuable insight for a future investigation and test of the role of promoter divergence in tree stress responses. We felt that this analysis adds novelty and depth to our study and addresses an important discussion point for future investigations as well as interpretation of our current experimental results on gene expression. Therefore, to the benefit of the reader, we decided to include these results in the main text and added as the new **Figure 3**.

7. The conclusion is well stated. You could strengthen it with one or two sentences about future applications, e.g., “The system can now be used to test microbial inoculants or cross-communication between tree genotypes.”

We appreciate this suggestion, and have added additional sentences to the conclusion (lines 447 - 451) addressing future applications of our research: ”This system offers a valuable platform for exploring the role of different microbial inoculants and the cross-communication between tree species or intra-specific genotypes. Specifically, it will facilitate investigations into the impact of pathogen attacks on trees interconnected by common mycorrhizal networks (CMN), helping to uncover the mechanisms behind CMN-mediated defense responses in forest ecosystems.”

**Reviewer #2:** 
1. Abstract states an aim to identify and validate known plant defense markers in aspen and birch, while Conclusion presents a methodological pipeline for homologue discovery. Please harmonize Abstract, Introduction, and Conclusion so the study goal and take-home message are consistent across sections.

We thank the reviewer for this valuable suggestion. In response, we have revised the Abstract and final paragraph of the Introduction to clearly harmonize the study aim and take-home message with the Conclusion. The revised text now emphasizes that the primary goal of the study is the identification and validation of specific plant defense markers in hybrid aspen and silver birch, with the in vitro propagated tree clone system presented as a reproducible framework enabling this analysis. Key results, including the identification of LOX2, MPK3, and EIN2 in birch and JAZ10 together with EIN2 in aspen, are now explicitly stated in the Abstract, ensuring consistency across all sections.

2. The combined treatment is wound plus 5 uL of 1 uM flg22 applied on the wound, while the control is water on an unwounded leaf. A wound plus water control is needed to isolate the added contribution of flg22 on a wound. If these data are not available, state this limitation clearly and moderate any claim of additivity or synergy.

We thank the reviewer for considering the controls for the combined treatment and acknowledge that this is a valid discussion point and would like to offer our view on the relevant comparisons and controls. The combined treatment of wound and flg22 may effectively involve simultaneous perception of DAMP and PAMP signals such as in the study by Pastor et al. (2022), <https://doi.org/10.1007/s00425-022-03918-y>.

In this study, the co-application of the bacterial peptide flagellin with the DAMPs (cyclic AMP or cellobiose) transiently amplifies PAMP-triggered ROS burst as well as RbohC gene expression. In all cases, application of a single elicitor (flg22, cAMP or cellobiose) was compared to the combined treatments and to individual water control for making the conclusions. With similar logic, we wish to argue that one of the reasons for expressing our data as log2FC in Figure 2 was to compare all treatments to the same reference, i.e., water control. For example, by comparing flg22 treatment to H20 and flg22+wounding to H2O, the only differential in the two pairwise comparisons is the presence of wounding in the second comparison. Therefore, while we have not directly used ‘’water on an unwounded leaf’’ as control for ‘’flg22 + wounding’’ treatment, we have effectively compared one variable at the time when normalizing all treatments against water control first. Another study on PTI responses in roots by Geldner group in Lausanne (<https://www.sciencedirect.com/science/article/pii/S009286742030060X>) demonstrated that mechanical damage by laser ablation is necessary for flg22-induced defence marker gene expression (FRK) in specific root sections (Figure 1 and Figure 2 of the study). Importantly, this experiment did not explicitly state using H2O as control in -flg22 treatments, except for Figure 3 where mock is used as control of ectopic DAMP application. Both studies cited above implicate that wound and flg22 elicited responses may interact and potentiate marker gene responses, which is the observation in our study as well. Moreover, another study in our group identified wounding + flg22 as strong reproducible inducer of systemic signals in Medicago truncatula leaves and was selected for downstream studies of mycorrhiza mediated inter-plant responses (<https://www.biorxiv.org/content/10.1101/2024.12.03.626652v1>) which we also aim to test in the system with both tree species presented here.

While we did not originally include “water+wound” control and unfortunately do not have yet capacity and resources to repeat all the experiments of our manuscript with extra control within the short time-frame, we agree with reviewer that we could include ‘’wound’’ and ‘’wound+water’’ control in our future experiments to verify whether water drop may modify the typical wound responses, e.g., via diluting the DAMPs released by wounded cells into 5uL volume or any other mechanism. Strictly speaking, we cannot disprove this and have carefully added this consideration in the discussion to reflect the point raised by the reviewer (lines 430-436). Nevertheless, we believe that we have indirectly circumvented the necessity for the “water+wound” control by comparing all treatments to the same H20 reference, for reasons described above, and therefore can make meaningful (albeit, admittedly, not absolute) conclusions from our data.

3. FLS2 perceives flg22 in the apoplast at the plasma membrane. A small surface droplet can have variable penetration through cuticle and stomata in in vitro leaves, which can explain weak flg22 alone effects, and wounding can enhance entry. Please justify the chosen delivery and either show that surface application gives reliable uptake such as a simple tracer or wetting validation and residence time, or consider a more reliable delivery such as gentle syringe infiltration in water or a validated spray with a minimal wetting agent, with matching mock controls. In all cases, include a wounded plus water control to isolate the added contribution of flg22. 1 uM is plausible yet responses are dose dependent and species dependent. If feasible, consider adding dose–response data, otherwise acknowledge this limitation. Early PTI peaks often occur within minutes up to about two hours. Please justify the focus on 4 h and 24 h and discuss likely missed early responses, and consider adding an early time point. Please provide a short rationale for using the combined stimulus that explains ecological co-occurrence and expected hormonal crosstalk, and please clarify whether the interaction was a planned hypothesis or exploratory. In the analysis, please report simple effects that distinguish combination effects from potential entry effects at the wound, for example, adding flg22 at a wound versus wounded plus water.

We acknowledge that the reviewer raised an important question with regards to penetration of flg22 droplets through the leaf surface. A scenario where the hydrophobic properties of leaf cuticle or closure of stomata may prevent the penetration of flg22 could introduce variation in the apoplastic concentration of the elicitor. Nevertheless, flg22 is known to show its activity within nM concentration range (e.g., starting from 10-2 nM, doi: 10.1046/j.1365-313X.1999.00265.x). In fact several studies reported 100nM and 10nM *flg22* to induce strong ROS burst (e.g., doi: [10.1111/nph.12408](https://doi.org/10.1111/nph.12408); doi: [10.5511/plantbiotechnology.22.0823a](https://doi.org/10.5511/plantbiotechnology.22.0823a); doi: 10.1038/s41467-024-54271-5). We therefore believe that 1uM concentration would still be relatively high to expect immunogenic properties even when diluted 2 to 3-fold during the penetration in the apoplast.

We reasoned that for proper entry to occur, firstly, the drop would need to adhere to the leaf surface and, secondly, the leaf should display detectable water uptake. To test this and empirically address the reviewer’s comment, we decided to measure the contact angle of the droplet as this can indicate how hydrophobic the leaf surface is. Aryal & Neuner (2010) (<https://link.springer.com/article/10.1007/s00442-009-1437-3>) have devised a method to measure the contact angle (*theta*) of a droplet onto the leaf surface to determine how wettable is the surface (see illustration below).

We took this approach and measured *theta* for 5uL of 1uM flg22 solution, and found that the median theta for aspen was 45° and the theta for birch was relatively higher - around 80° (Supplemental Figure 4A) suggesting ‘’wettable’’ properties in both species. To visualise the behaviour of a droplet immediately after deposition onto the surface, we additionally took several representative macro videos which can be reviewed via this google drive link [Surface angle videos](https://drive.google.com/drive/folders/1L61-_p28hl0d2bUzC4GjZzrWsFckuWCS?usp=sharing). By observing the droplet behaviour, we were convinced that the applied liquid instantly adheres to the aspen leaf surface, corresponding to theta around 30-40°. The droplet in a birch, however, displayed more pronounced droplet formation with estimated theta to be closer to 90° which was corresponding to the measured *theta* from macro photographs of an independent set of leaves, displayed in Supplemental Figure 4A.

Next, we additionally took the approach and the exact method by Limm et al. 2009 (<https://link.springer.com/article/10.1007/s00442-009-1400-3>) to determine leaf water uptake through leaf blade in 3 hours, corresponding to the timing prior our 4h sampling in **Figure 2**. We performed this experiment with water, rhodamineB solution as well as rhodamineB solution with added surfactant Tween80. RhodamineB is a water soluble mobile dye, commonly used in xylem flow measurements and liquid tracing in plant tissues. Intriguingly, both tree species displayed significant leaf apoplastic water saturation independent of rhodamineB and surfactant addition (Supplemental Figure 4B), suggesting that water-based solutions may penetrate the leaf of hybrid aspen and silver birch very well. Notably, the surfactant addition did not significantly increase water absorption into the leaf. This further corroborates the importance of foliar water uptake (FWU) in trees (as review by Schreel & Jeroen, 2020; 10.1016/j.tplants.2020.01.003). To complement these results, we also applied a single 5 uL drop of rhodamineB solution to the abaxial side to observe the staining after 3h under the light microscope with crude longitudinal leaf blade cross-sections (Supplemental Figure 4C). Aspen displayed very intense staining of mesophyll tissues with rhodamineB solution. In comparison, the birch leaves displayed relatively patchy staining across longitudinal sections of leaf blade but had very intense colouring within each patch. Intriguingly, closer inspection of stomatal openings on birch epidermis indicated that stomata could be prime entry points of rhodamineB into the leaves and explain the patchy staining beneath the stomatal sites.

Taken together, the experiment above collectively suggest that flg22 may penetrate and could activate immunity in both hybrid aspen and silver birch, especially given the reported nM range activity flg22 in ROS assays by other studies.

4. ACT2 was chosen because it was amplified earlier and looked uniform compared with TUB, SAND, and GAPDH. This alone does not establish stability. Evaluate at least two candidate reference genes with a standard stability method and either use two gene normalization or show ACT2 stability with no treatment effect on Ct in the same conditions. Reference gene stability can be demonstrated by flat ACT2 Ct across water, flg22, wound, and combination within each time point. Also, state clearly in the main text and in figure legends the replication structure.

We acknowledge that we can improve clarity about describing reference selection by adding more detail about the choice of the reference gene. We have provided additional explanation how *ACT2* was selected in lines 240-251, whereby we state that in addition to early amplification displayed in Supplemental Figure 4, *A*CT2 ‘’did not display any significant differences in amplification cycle across water, flg22, wound and wound+flg22 treatments in silver birch at 4h (F=1,245 ; p=0,337) and 24h (F=0,64912 ; p=0,6101) or hybrid aspen at 4h (F=1,247; p=0,336) and 24h (F=1,9681; p=0,2345)’’. While we agree with the reviewer that inclusion of multiple reference genes may be beneficial, this implicates much higher costs in terms of additional reagent consumption and labour time, simultaneously consuming more cDNA that may be scarce when total RNA yields are low in the experiments and new cDNA cannot be acquired without harvesting more plant material. Also, too diluted cDNA may obscure measurements of some low level transcripts. Unless justified, for practical reasons using a single reference gene would be the preferred strategy. Nevertheless, to address the suggestion to use two reference genes, we randomly selected two genes and stress treatments (along with water control) for both tree species and repeated qPCR and normalized the gene expression with geometric mean from 2 reference genes to compare the normalized expression when using a single gene reference. The results in **Supplemental Figure 6** suggest that the additional reference may not change the trend for gene induction or suppression in response to stress. Guided by this result we did not opt for inclusion of two reference genes for all other comparisons as this would require to completely redo the entire rt-qPCR measurements displayed within the manuscript to be consistent that the two reference genes are run on the same plate as the test gene at all times.

As requested by the reviewer, we have included an explanation about the qPCR plate setup and replication structure in figure legends. For example, Figure 1 legend now states: ‘’Ct values were calculated as average from 3 technical replicate measurements for each sample. Gene expression was normalized to ACT2 as reference (ΔCt). Three test genes and ACT2 reference were measured together in the same qPCR run for 4 independent biological replicates of at least 2 treatments.

5. The expression boxplots in Figure S4 show distributions and post hoc groups and thus improve interpretability. Promote them to a main figure next to the heatmap. Add n on each panel.

We thank the reviewer for the suggestion and display the number of samples used in treatment comparisons for each gene in **Supplemental Figure 4** and the legend. We have promoted the boxplot of EIN2 expression to the new Figure 1 along with the phylogenetic tree that was complimentary suggested by the reviewer 1. The new Figure 1 logically tells a story about the methodological approach in identifying tree stress gene homologues using EIN2 as an example for all other marker genes tested in our study. This is then followed by fold change analysis of all stress responsive markers in the main **Figure 2** (previously **Figure 1**) as well as potential primer divergence of selected stress markers as **Figure 3**. These changes collectively satisfy comments #4 & #6 by reviewer #1 as well as comment comment # 5 from reviewer #2. We considered moving the entire section of boxplots underneath the heatmaps but concluded that this would render each boxplot too small for good readability and the overall image too large and cluttered. The same applied to moving the entire set of phylogenetic trees. Instead, we decided that a better approach would involve promotion of results from a single gene as an example and direction to the supplemental figures for the entire list of genes tested.

6. IBA is present in culture. Auxin can influence gene expression. Please justify its inclusion and discuss any potential confounding of defense marker dynamics.

As described in the methods, auxin was added to the growth medium, following a standard procedure for clonal propagation of trees in tissue culture facility of the the Latvian Forest Research Institute *Silava*, and is reported in previous studies (e.g., <https://doi.org/10.3390/plants11202692>). Auxin aids rooting of the cuttings and is therefore necessary to propagate and generate the material for the experiment. The reviewer is right to point out that auxin may display crosstalk with stress responses controlled by other plant hormones such as SA. For example, auxin may promote virulence of *Pseudomonas syringae* by suppressing SA responses (<https://doi.org/10.1094/MPMI-02-20-0047-R>; <https://doi.org/10.1111/tpj.12157>). To acknowledge the possibility of such hormonal crosstalks, we have added this point to the discussion (line ) and cited the references in the text.

7. Consider avoiding phrases like strongest response or good or useful or strong marker. Please state the measured change for each claim with its interval and adjusted p value. Please define a simple criterion for using the word marker and apply it consistently.

We thank the reviewer for the suggestion. We have critically evaluated the description of our results and, guided by the reviewer’s comment, decided to delete the adjectives ‘’good’’, ‘’strong’’, ‘’useful’’ as this may add a level of subjectivity and ambiguity. To support claims about the reported induction or suppression of a particular stress responsive gene, we have additionally stated the fold change values (+/-SEM) as well p-values in brackets after genes in the results section. Moreover, we agree with the reviewer that the term ‘’[molecular] marker’’ was used loosely at times and could in fact also mean other molecular read-outs such as ROS burst, Ca2+ waves, electrical signal etc. To avoid such confusion, we have either deleted the ‘’marker’’ or substituted the term ‘’marker’’ with ‘’stress responsive genes’’ or similar. for more precise meaning. We kept the term ‘’marker genes’’ when we reviewed literature and reported well established JA or SA pathway responses from studies in Arabidopsis.

8. Only one clone per species was tested. Frame results as clone level patterns and putative markers to be validated across more genotypes and growth conditions including soil grown plants. Revise the Conclusion accordingly.

Indeed, we analyzed a single commercially grown and high yielding clone from each tree species. In order to establish the stress responsive genes as representative markers for a particular tree species, future studies should additionally evaluate the gene responses across diverse genotypes of silver birch and aspen hybrids. To acknowledge the point, we included additional sentences to conclude the discussion (lines 507-511).

9. Add the following to Supporting Information. Include the exact amplicon length for each primer pair. Report the standard curve slope and R squared used to calculate amplification efficiency together with the dilution range already stated. Provide raw Ct or delta Ct tables for all samples. Provide basic RNA integrity information such as RIN values or gel traces. Deposit the R code used for data processing, statistics, and figure generation in a public repository with a persistent identifier.

We thank the reviewer for suggesting inclusion of additional supporting information in our manuscript. We have included exact amplicon length for each primer pair, available as **Supplemental table 1**. We have included the new **Supplemental Figure 5** displaying Ct across dilution series used to calculate the slope, R2 as well as primer efficiency for each target gene. We have included **Supplemental Table 2** with the source data and ΔCt as well as ΔΔCt values used to generate the boxplots and heatmaps. We have added the R source code for statistical analysis as **Supplemental File 2,** freely available together with our manuscript. We have updated the methods section to report that we initially used Nanodrop for quantifying total RNA and determining sample purity (260/280 and 260/230 ratios) and did not perform any additional RNA quality check before cDNA synthesis. Instead, we only used samples with total RNA > 50 ng/uL and 260/280 and 260/230 ratios between [1.8-2.2]. We would usually perform the Bioanalyzer for library construction for RNA-seq experiment but not rt-qPCR on dozens of samples due to reagent costs and availability. Nevertheless, we wished to reassure the reviewer that we have no reason to believe that the RNA could have been unsuitable for qPCR. We were kindly offered a few available slots of a HS total RNA chip on Agilent 4200 Tapestation by our colleagues at Genome Centre who were conducting library preparations at the time of this revision. We put a small subset of our samples to visualize 28S and 18S rRNA bands (triangles) and include the electrophoresis gel below. Please, note that this was done approximately 1 year after the extraction of the original total RNA and may not represent the RNA integrity at the time of the cDNA synthesis which immediately followed the extraction step. For this reason we were reluctant to rerun all our samples on the Bioanalyzer now and resorted to rechecking a small subset of samples from both tree species added below.

Minor comments

- Fix typos and inconsistencies. Chinchilla spelling. LOX2 not LOC2. CoGe not GOGE. Remove the duplicate Louis and Shah 2015 reference.

The errors pointed out have been fixed.

- Phylogeny figures need higher legibility. Export higher dpi or vector graphics. Add a short note on how the final homolog was chosen when several paralogs cluster near Arabidopsis or Medicago.

Figures exported in higher (500) DPI. A short explanation on how certain homologues were chosen when multiple potential candidates clustered near Arabidopsis and Medicago sequences was added to the figure legend.

- Regarding primer specificity, include representative melt curves for each target in Supporting Information and annotate band sizes on gels in Figure S3 or list the sizes in Table S1.

Representative melting curves for all reported genes were added as Supplemental Figure 3.

- The study focuses on eight genes including LOX2 and JAZ10 for wound and JA signaling, and MPK3 and EIN2 for cross talk. Please explain how the candidate list was narrowed to these eight and why other widely used wound markers were not included such as AOS, AOC, OPR3, VSP1 or VSP2, COI1, JAR1, MYC2, or protease inhibitor genes. If additional homologues were screened in silico, list them in Supporting Information with brief reasons for exclusion. This will help readers understand the design scope and calibrate conclusions.

We provide a rationale for investigating wound and flg22 responsive genes in JA and SA pathways respectively, naming the corresponding genes of our study in lines 96-100 of introduction. For clarity and benefit of the reader, we list all 8 tested genes in the methods section 2.4 and refer to **Supplemental Table 1** where we provide gene IDs, primer sequences and referenced explanation for gene function of MPK3, EIN2, PAD4, MYB51, FRK1, LOX2, NHL10 in plant stress responses, as requested. Furthermore, we discuss the known functions of the abovementioned genes in discussion section 4.2.

We agree with the reviewer that many other genes might have been additionally included to represent JA, SA, ET and other phytohormone pathways as well as their numerous crosstalks. Nevertheless, in qPCR study this will always be arbitrary selection of a limited number of genes (limited by time and resources to test them) compared to alternative approach with RNA-seq analysis which could be used in an unbiased stress responsive gene selection but would require qPCR verification downstream. Our work provides a methodological approach on how to identify and characterise any response gene of interest and our primary aim was not to include an exhaustive list of all wound markers but to establish an experimental system enabling subsequent screening for a range of different defence elicitors and their responses in future studies.
