# Supplementary material for: Species specific marker genes for systemic defence and stress responses to leaf wounding and flagellin stimuli in hybrid aspen and silver birch
Source: PLoS One. 2026 Mar 12;21(3):e0344803. doi: 10.1371/journal.pone.0344803 (PMC12981503; doi:10.1371/journal.pone.0344803)
Supplement: Attachment — Submitted filename: Respone to reviewers 2_PlosOne_Feb_2026.docx [file pone.0344803.s017.docx]

**PONE-D-25-43089R2**

Species specific marker genes for systemic defence and stress responses to leaf wounding and flagellin stimuli in hybrid aspen and silver birch

Dear editor,

We are grateful for careful consideration and evaluation of our revised manuscript **PONE-D-25-43089R2** for publication in *PlosOne*. Following the feedback and comments from the reviewers and editor, we here resubmit our revised manuscript with the following corrections in response to the requested changes:

1. ***Include references to online tools and resources.***

We have included the missing references in materials & methods section and the reference list:

*JASPAR*

Baydar Ovek D, Rauluseviciute I, Aronsen DR, Blanc-Mathieu R, Bonthuis I, de Beukelaer H, Ferenc K, Jegou A, Kumar V, Lemma RB, Lucas J, Pochon M, Yun CM, Ramalingam V, Deshpande SS, Patel A, Marinov GK, Wang AT, Aguirre A, Castro-Mondragon JA, Baranasic D, Chèneby J, Gundersen S, Johansen M, Khan A, Kuijjer ML, Hovig E, Lenhard B, Sandelin A, Vandepoele K, Wasserman WW, Parcy F, Kundaje A, Mathelier A JASPAR 2026: expansion of transcription factor binding profiles and integration of deep learning models Nucleic Acids Res. 2026 Jan 6;54(D1):D184-D193.; doi: 10.1093/nar/gkaf1209

*PlantPan*

Chow CN, Yang CW, Wu NY, Wang HT, Tseng KC, Chiu YH, Lee TY, Chang WC. 2024. PlantPAN 4.0: updated database for identifying conserved non-coding sequences and exploring dynamic transcriptional regulation in plant promoters. Nucleic Acids Research 52: D1569–D1578.<https://doi.org/10.1093/nar/gkad945>

*PlantGenIE*

Sundell D, Mannapperuma C, Netotea S, Delhomme N, Lin YC, Sjödin A, Van de Peer Y, Jansson S, Hvidsten TR, Street NR. 2015. The Plant Genome Integrative Explorer Resource: PlantGenIE.org. New Phytologist 208: 1149–1156.<https://doi.org/10.1111/nph.13557>

*CoGe*

Lyons E, Pedersen B, Kane J, Alam M, Ming R, Tang H, Wang X, Bowers J, Paterson A, Lisch D, Freeling M. 2008. Finding and comparing syntenic regions among Arabidopsis and the outgroups papaya, poplar, and grape: CoGe with rosids. Plant Physiology 148: 1772–1781. https://doi.org/10.1104/pp.108.124867

*TAIR*

Reiser L, Bakker E, Subramaniam S, Chen X, Sawant S, Khosa K, Prithvi T, Berardini TZ. 2024. The Arabidopsis Information Resource in 2024. Genetics 227: iyae027. https://doi.org/10.1093/genetics/iyae027

2. ***Please also make sure to align figures so that the same order of model system (Birch and hybrid-aspen) occur in the figure and in the legend - this is relevant for figures 1 and 2.***

We have updated Figures 1 and 2 to make sure that *Populus tremula x tremuloides* data are displayed in panel A of both Figure 1 and 2, while *Betula pendula* data in panel B of the Figures 1 & 2. We made sure that figure legends list the species correspondence to the panels accordingly.

3. ***In Figure 1 for EIN2 in the hybrid 24h - the authors should carefully review p-value and post hoc letters ... one or the other must be incorrect as there are only A, and ABs and no Bs in the boxplot.***

We thank the reviewer for noticing the discrepancy. We have corrected the post-hoc test letters in Figure 1 and Supplemental figure 7.

4. **In Figure 2 in which the problem re-occurs with different order of birch and poplar mentioned in the legend and presented in the figure. In addition, the heat maps follow different rankings so neither treatment nor gene occur in the same order in the heatmap clusters. This difference in orders should be emphasised in the figure legend, or alternatively the heatmaps could be reorganised with fixed ranking of treatment and gene.**

We have updated Figures 1 and 2 to make sure that *Populus tremula x tremuloides* data are displayed in panel A of both Figure 1 and 2, while *Betula pendula* data in panel B of the Figures 1 & 2. We made sure that figure legends list the species correspondence to the panels accordingly. In Figure 2, we have removed the hierarchical treatment clustering to give the same order of the treatments and genes in both time-points and tree species.

For the ease of tracking changes in the manuscript, we include the file *Track changes.docx.* **The final version** with all track changes accepted is also enclosed and uploaded to the online submission system. We would like to update our funding statement as follows: ‘’This work was supported by Latvian Council of Science Fundamental and Applied Research grant lzp-2022/1-0283. The funders had no role in study design, data collection and analysis, decision to publish, or preparation of the manuscript.’’ We have deleted funding statement from the manuscript file.

We hope that the improvements presented herein will be sufficient to address the points raised by the reviewers. We are looking forward to your final decision.

Sincerely,

Dr. Zigmunds Orlovskis
